# A *Wickerhamomyces anomalus* Killer Strain in the Malaria Vector *Anopheles stephensi*

**DOI:** 10.1371/journal.pone.0095988

**Published:** 2014-05-01

**Authors:** Alessia Cappelli, Ulisse Ulissi, Matteo Valzano, Claudia Damiani, Sara Epis, Maria Gabriella Gabrielli, Stefania Conti, Luciano Polonelli, Claudio Bandi, Guido Favia, Irene Ricci

**Affiliations:** 1 Scuola di Bioscienze e Medicina Veterinaria, Università degli Studi di Camerino, Camerino, Italy; 2 Dipartimento di Patologia Animale, Igiene e Sanità Pubblica Veterinaria, Università degli Studi di Milano, Milan, Italy; 3 Dipartimento di Scienze Biomediche, Biotecnologiche e Traslazionali, Università degli Studi di Parma, Parma, Italy; Centro de Pesquisas René Rachou, Brazil

## Abstract

The yeast *Wickerhamomyces anomalus* has been investigated for several years for its wide biotechnological potential, especially for applications in the food industry. Specifically, the antimicrobial activity of this yeast, associated with the production of Killer Toxins (KTs), has attracted a great deal of attention. The strains of *W. anomalus* able to produce KTs, called “killer” yeasts, have been shown to be highly competitive in the environment. Different *W. anomalus* strains have been isolated from diverse habitats and recently even from insects. In the malaria mosquito vector *Anopheles stephensi* these yeasts have been detected in the midgut and gonads. Here we show that the strain of *W. anomalus* isolated from *An. stephensi*, namely *Wa*F17.12, is a killer yeast able to produce a KT in a cell-free medium (*in vitro*) as well as in the mosquito body (*in vivo*). We showed a constant production of *Wa*F17.12-KT over time, after stimulation of toxin secretion in yeast cultures and reintroduction of the activated cells into the mosquito through the diet. Furthermore, the antimicrobial activity of *Wa*F17.12-KT has been demonstrated *in vitro* against sensitive microbes, showing that strain *Wa*F17.12 releases a functional toxin. The mosquito-associated yeast *Wa*F17.12 thus possesses an antimicrobial activity, which makes this yeast worthy of further investigations, in view of its potential as an agent for the symbiotic control of malaria.

## Introduction

*Wickerhamomyces anomalus* (formerly *Hansenula anomala* and *Pichia anomala*) is a *Saccharomycetes* yeast with wide biotechnological potential [1], traditionally used in the agro-food sector, as a biopreservation agent, suitable to improve feed and food safety. The suitability of *W. anomalus* as a biopreservation agent is certified by the European Food Safety Authority (EFSA), with the attribution of a qualified presumption of safety status at level-1 (QPS-1) [2]. Fields of application for this yeast include biocontrol, food fermentation, biofuel production, and the production of therapeutic molecules used in human medicine.

*W. anomalus* is highly tolerant to environmental stresses, and is adapted to a wide range of growth conditions, in terms of temperature (3–37°C), pH value (2–12) and osmolarity [3]. This robustness makes this yeast highly competitive in many different habitats. It has been isolated from very diverse sources, including flowering plants, fruit skins, dairy and baked food products, contaminated oil, salted foods, wastewater, marine environments, human tissues and even the gut of insects (flies, beetles and mosquitoes) [1].

An additional character displayed by *W. anomalus* is a wide spectrum antimicrobial activity (something very unusual in yeasts), being active against a variety of microorganisms including other yeasts, filamentous fungi and bacteria [1]. Several inhibition mechanisms have been proposed such as competition for nutrients, production of ethyl acetate and direct killing by Killer Toxins (KTs). The yeast “killer” phenomenon derives from the lethal activity of KTs secreted by self-immune killer strains on sensitive yeasts, expressing specific cell-wall KT receptors (KTRs) [4].

KTs are a group of glycoproteins with a variable molecular weight, whose activity is characterized by a large range of optimal pH and temperatures [5]. These antimicrobial properties make *W. anomalus* a valuable bio-control agent on different substrates; for example the inhibition of the fungus *Botrytis cinerea* on fruits [6], [7], and of *Penicillium* spp., *Aspergillus* spp. and *Enterobacteriaceae* on cereal grain [8]. The mechanism of bacterial inhibition is yet unknown, but it is likely that *W. anomalus* KTs (*Wa*KTs) play a main role, as demonstrated on different Gram-negative bacteria and fungi [9], [10]. Killer activity mediated by *Wa*KTs has been well characterized against sensible strains of fungi. For example, *Wa*KT-mediated inhibition was demonstrated in *Candida albicans* both *in vitro* and *in vivo* [11]–[13]. Other studies highlighted that a KT-derived ‘killer’ peptide (KP), characterized by a candidacidal activity, aggregates on the surface of *C. albicans* cells, particularly on budding scars, where β1,3-glucans (the major component of the KTRs) are exposed [14]. KP has also been shown to be effective against *Saccharomyces cerevisiae*, including some strains resistant to conventional antifungal drugs [15], and against a wide spectrum of microbes beyond bacteria and fungi, including the protozoan parasites *Leishmania infantum*, *L. major* and *Acanthamoeba castellanii* [16], [17].

The mechanism of action of *Wa*KTs is mainly based on the interaction with cell-wall carbohydrates, where β-glucanase-induced hydrolysis of glucan into glucose is demonstrated [18]. In this context, several authors have investigated the β-glucanase activity of *Wa*KTs from different *W. anomalus* strains. In the strain K (successfully applied as a bio-control agent against *B. cinerea* on apple) and in killer strains isolated from olive brine, exoglucanase-encoding genes are responsible for the toxic exo-β-1,3-glucanase activity [19], [20]. The same *Wa*KT-mediated enzymatic activity in the marine *W. anomalus* strain YF07b is directed against pathogenic fungi of a crab [21]. Thus, arthropod-associated *W. anomalus* could exert a protective function against pathogens, in addition to the already demonstrated nutritional role (such as the case of the mutualistic yeast-beetle association with a nutritional dependence in the coleopteran *Doubledaya bucculenta* [22]). Among insects, *W. anomalus* has also been detected in the inner body of *Drosophila* sp.[23] and in mosquitoes of public health importance [24]. Particularly, it has been shown to be associated with the malaria vector *Anopheles stephensi*, where the yeast is present in the midgut as well as in the gonads [25].The association between *Anopheles* mosquitoes and *W. anomalus* deserves to be further investigated, in order to explore the possibility that this yeast could be developed as an agent for the symbiotic control (SC) of malaria [26], in addition to other candidates for SC, such as the bacteria *Asaia* spp. and *Wolbachia*, and the fungus *Metarhizium robertsii* [27]–[29].The objective of this work is to investigate whether the *W. anomalus* strain isolated from *An. stephensi* displays killer activity against model microorganisms, and whether it produces a KT antimicrobial molecule within the mosquito body.

## Materials and Methods

### Yeast Strains

Five strains belonging to the yeast species *W. anomalus*, *C. albicans* and *S. cerevisiae* have been used: (i) *Wa*F17.12 isolated from *An. stephensi* mosquitoes [25], (ii) *Wa*ATCC 96603, (iii) *Wa*UM3, (iv) *Ca*ATCC 24433 and (v) *Sc*ATCC 2601. *Wa*ATCC 96603 and *Wa*UM3 were used as reference strains, since they are respectively *Wa*KT-producing (*Wa*KT-resistant) and *Wa*KT-non-producing (*Wa*KT-susceptible) strains [30], [31]. The strain *Wa*UM3 was used both as negative control in the experiments aimed at studying the expression of *Wa*KT and susceptible target in antimicrobial assays. *Ca*ATCC 24433 and *Sc*ATCC 2601 were selected as additional *Wa*KT-susceptible targets [32]–[34].

### Mosquitoes

The experiments were performed using a colony of *An. stephensi* (Liston) reared at University of Camerino (Italy). Mosquitoes were maintained at standard aseptic conditions of 30°C and 95% humidity. Larvae were grown in tanks filled with water containing sterile minced commercial mouse food. The adults were fed with 5% sterile sugar solution. Specific diet for IFA assays is described in a paragraph below.

### Production of Crude WaKT Extracts

The strains *Wa*F17.12, *Wa*ATCC 96603 and *Wa*UM3 were grown in culture conditions suitable to stimulate the production of soluble toxins in liquid medium, as reported by İzgü et al. (2006) [33]: YPD broth (20 g/l peptone, 20 g/l glucose, 10 g/l yeast extract), buffered at pH 4.5 or pH 8 with 0.1 M citric acid and 0.2 M K_2_HPO_4_, and incubated at 26°C for 36 h at 70 rpm [32]. The yeast cells were removed by centrifugation at 2000 g 4°C for 10 min. The supernatants were filtered and concentrated 100-fold using Pierce Concentrators (Thermo Fisher Scientific Inc, Waltham, Massachusetts, USA) with a cut off of 9 kDa, by centrifugation at 4°C for 5 h at 2000 g.

### Western Blot Analysis of WaKT Crude Extracts

The crude *Wa*KT extracts from *Wa*F17.12, *Wa*ATCC 96603 and *Wa*UM3 were analyzed by electrophoresis in 7% polyacrylamide gel, at 100 Volts for 4 h in a minigel system (Bio-Rad Laboratories, Richmond, Calif., USA). Proteins were electrically transferred to a polyvinylidene difluoride (PVDF) membrane at 30 Volts overnight. PVDF membrane was incubated for 1 h at room temperature with 3% non-fat powdered milk in phosphate-buffered saline (PBS) at pH 7.2 and 2% tween-20 (PBS-T). Subsequently, the membrane was incubated at 37°C for 1 h with a 1∶50 dilution of monoclonal antibody (mAb) KT4 produced against a *Wa*KT of *W. anomalus* ATCC 96603 [14], [30] in PBS-T, washed three times in PBS-T and incubated for 1 h at room temperature with a secondary, peroxidase-conjugated, anti-mouse Ab. After three washings with PBS-T, the membrane was incubated 1 min with the proper substrate (Ablotlite, Euroclone, Italy) and detected by ChemiDoc 2000R (Kodak, USA).

### WaKT Activity Assays

The tests to evaluate the toxic activity of *Wa*F17.12 on susceptible yeast strains were preliminary performed using supernatants from *W. anomalus* cultures against *S. cerevisiae*. *Wa*F17.12, *Wa*ATCC 96603 (positive control) and *Wa*UM3 (negative control) were grown at conditions that stimulate *Wa*KT production, as previously described (see paragraph on the *Production of crude WaKT extracts*), at pH 4.5. Then the three supernatants were filtered and used as growing-media for the yeast strain *Sc*ATCC 2601. *S. cerevisiae* was inoculated in fresh YPD medium pH 4.5 for normal growth control. After an overnight incubation at 26°C and 70 rpm, optical densities (OD) were measured by a NanoDrop 1000 Spectrophotometer (Thermo Fisher Scientific Inc, Waltham, Massachusetts, USA).

An antimicrobial assay was carried out on solid medium plate against *Asaia* sp. (α-proteobacterium, Gram-negative), isolated from *An. stephensi* mosquito [27]. An overnight *Asaia* culture, grown in liquid GLY medium (25 g/l glycerol, 10 g/l yeast extract, pH 5) at 30°C at 100 rpm, was diluted to 0.1 OD (10^7^ cells/ml) in sterile water and seeded on GLY agar plate (25 g/l glycerol, 10 g/l yeast extract, 20 g/l agar pH 5). In accordance with Lopes et al. (2010) [35], five microliters of three dilutions (10^9^, 10^8^ and 10^7^ cell/ml) of a *Wa*F17.12 yeast culture, grown in stimulating conditions, were spotted in duplicate and the plates were incubated for 72 h at 20°C. Five microliters of a *Wa*UM3 culture (10^9^ cell/ml) were spotted as negative control. As positive control, the same experiment was performed seeding *Sc*ATCC 2601 (susceptible strain) on a YPD plate (10 g/l yeast extract, 20 g/l bacterial peptone, 20 g/l glucose, 20 g/l agar, 0.03 g/l methylene blue). After 72 h, plates were observed for the presence of inhibition halo around the spots.

Further killer activity tests of strain *Wa*F17.12 were carried out against the susceptible strains *Ca*ATCC 24433 and *Wa*UM3, using *Wa*KT crude extracts obtained from *Wa*F17.12, *Wa*ATCC 96603 (positive control) and *Wa*UM3 (negative control) at different pH conditions (4.5 and 8); these *Wa*KT crude extracts were prepared as described above. The sensitive strains were grown overnight in YPD medium buffered at pH 4.5 (*Ca*ATCC 24433 or *Wa*UM3) or pH 8 (*Wa*UM3) with 0.1 M citric acid and 0.2 M K_2_HPO_4_. Cells (10^5^ per well) of each susceptible strain were spotted in a 96-well microtiter plate and incubated with the three KT crude extracts at a final concentration of 50X or 19X in YPD medium. To control the normal growth of the susceptible strains, cells were incubated with fresh YPD and free medium was spotted in additional wells to rule out a possible environmental contamination. After 12 h of incubation at 26°C, the toxic activity of crude extracts against the susceptible strains was evaluated by checking cell morphology, cell density (established by OD measure) and by analysing the cellular vitality through a trypan blue assay on a Neubauer counting chamber using a 40X optical microscope objective (Carl Zeiss Axio Observer.Z1, Milan, Italy).

### Statistical Analysis

All the above assays with crude extracts (50X or 19X) on the susceptible strains CaATCC 24433 and WaUM3 were performed in triplicates and vital cells/ml were counted twice. The analysed data are thus those that derived from three cell counts for each assay (using the means of the two counts for each assay). The data from the cell counts were analyzed using the software GraphPad (available at http://www.graphpad.com/) and statistical analysis was performed using One Way ANOVA followed by Bonferroni’s Multiple Comparison Tests. Statistical significance is expressed as a p-value, p<0.05.

### Analysis of WaKT Expression by In vitro and In vivo Immunofluorescence Assays (IFA)

*Wa*KT expression was tested by IFA using mAbKT4 both *in vitro* on cultivated yeasts and *in vivo* within the mosquito body. Strains *Wa*F17.12, *Wa*ATCC 96603 (positive control) and *Wa*UM3 (negative control) were grown at the conditions that stimulate toxin production (see paragraph on the *Production of crude WaKT extracts*), at pH 4.5. Cells were then centrifuged at 2000 g for 10 min at 4°C and washed twice in PBS 1X, for the *in vitro* assay, or three times in 9 g/l NaCl and resuspended in 50 g/l sucrose solution at a concentration of 10^8^ cells/ml to be added to the diet of newly emerged adult mosquitoes, for *in vivo* assay. In the latter experiment four cages were prepared containing mosquitoes fed with: i) sterile sugar solution, ii) sugar solution added with stimulated *Wa*ATCC 96603 cells, iii) sugar solution added with stimulated *Wa*F17.12 cells or iv) sugar solution added with stimulated *Wa*UM3 cells. After two days of feeding, the mosquitoes were moved in new cages and fed with sterile sucrose solution only. Hence, 10, 20, and 27 days post yeasts introduction mosquitoes were analyzed. Around 60 organs (midguts and gonads) were dissected and analyzed per each time point (total 171).

For IFA, cultivated yeast cells and dissected mosquito organs were fixed on slides with 4% paraformaldehyde for 10 min at 4°C and washed twice with PBS 1X. Organs were permeabilized with 0.5% Triton X-100 in PBS 1X for 10 min at room temperature and washed three times with PBS 1X. The slides were incubated in 1% BSA in PBS 1X for 30 min at room temperature and, successively, for 1 h at 37°C in mAbKT4 diluted 1∶1000 in PBS 1X. After three washings in PBS 1X, they were incubated for 30 min at 37°C with anti-mouse IgG Alexa Fluor 594 conjugate (Invitrogen, Life Technologies) diluted 1∶100 in PBS 1X and washed three times for 10 min with PBS 1X. Slides were mounted with glycerol and visualized by an epifluorescent microscope (Carl Zeiss Axio Observer.Z1, Milan, Italy).

### Immunohistochemistry

Five mosquitoes from cages i, ii, iii and iv were analyzed for the immunolocalization of *Wa*KT. The abdominal segments were excised and immediately fixed in 4% buffered formalin solution for 2 h at room temperature. After dehydration in a graded ethanol series, the samples were cleared in xylene and embedded in paraffin at 56–58°C. Sections (5 micron thick) were mounted on gelatin-coated slides, rehydrated and processed for immunohistochemical staining using the Vectastain ABC kit reagents (Vector Laboratories, Burlingame, CA). After inactivation of the endogenous peroxidase (0.3% H_2_O_2_ in methanol for 30 min) and blocking of endogenous avidin-binding activity (avidin-biotin blocking kit; Vector Laboratories), sections were incubated for 30 min in normal goat serum diluted 1∶5 with 1% bovine serum albumin (BSA; Sigma, St. Louis, MO) in 0.05 M phosphate-buffered saline (PBS), pH 7.6. Incubation of sections with the monoclonal antibody mAbKT4 was performed at dilutions from 1∶100 to 1∶2,000 in PBS containing 1% BSA overnight at room temperature, in a humid chamber. After washing, sections were incubated with goat biotinylated anti-mouse IgG (1∶200 in PBS) for 45 min, followed by washing in PBS and treatment with the avidin-biotin-peroxidase complex (1∶100) for 45 min. After washing in PBS, the immunoreactive polypeptides were visualized in all sections by incubation for 4 min with VIP substrate (Vector Laboratories) and the reaction was stopped by rinsing with tap water. Finally, sections were dehydrated and mounted in Eukitt. Immunohistochemical controls were carried out by incubation with preimmune serum or with PBS plus 1% BSA, in place of the primary antibody. Analysis has been performed using a photomicroscope Leica DM2500.

### Cloning and Sequencing of WaF17.12exoglucanase-encoding Genes (EXG1 and EXG2)

Genomic DNA was extracted from a culture of the strain *Wa*F17.12 using a JetFlex kit (Genomed, Germany). Amplifications of *EXG1* and *EXG2* genes were obtained using specific oligonucleotides (described in **Table S1**) designed on the *W. anomalus* BS91 *EXG1* and *EXG2* sequences available in the data bases (accessions: JQ734563 and JQ734566). Reaction mixtures were prepared in 25 µl using: 0.6 U of Dream Taq Polymerase (Fermentas, Thermo Fisher Scientific Inc, Waltham, Massachusetts, USA), 0.25 mMdNTPs, 1X Taq Polymerase Buffer, 0.2 µM each primer and 50 ng DNA. Reactions were run for 2 min at 95°C and cycled 30 times through 30 sec at 95°C, 30 sec at 55°C and 40 sec at 72°C. Finally, reactions were kept for 8 min at 72°C. PCR products were then resolved in 1% agarose gel stained with ethidium bromide. The fragments were cloned in T-Vector System following manufacture’s instructions (Promega, USA). The cloned fragments were sequenced after colony PCR using the plasmid primers SP6 (5′-atttaggtgacactatagaat-3′) and T7 (5′-aatacgactcactataggg-3′). The obtained sequences were firstly analysed by BLASTN. (http://blast.ncbi.nlm.nih.gov/Blast.cgi). Sequence alignment were then generated by ClustalW (http://www.genome.jp/tools/clustalw/). Sequences of *Wa*F17.12 genes *EXG1* and *EXG2* were deposited through the EMBL-Bank.

## Results

### Characterization of WaF17.12-KT Using Monoclonal Antibody KT4 (mAbKT4)

Western blot analysis was carried out on *Wa*KT crude extracts from the strains *Wa*F17.12 (from *An. stephensi*), *Wa*ATCC 96603 (*Wa*KT-producing strain) and *Wa*UM3 (*Wa*KT-non-producing strain) cultivated at a growth condition that stimulates toxin production [32], [33]. The monoclonal antibody (mAbKT4) used for labelling was directed against a specific *Wa*KT, produced by the reference strain *Wa*ATCC 96603 and well characterised for its wide antimicrobial activity; mAbKT4 has already been shown to cross-react with the toxin produced by other strains of killer yeasts [14], [30]. Western blot analysis revealed a single band of the same size in both *Wa*F17.12 (mosquito strain) and *Wa*ATCC 96603 (positive control) KT crude extracts; no signal was present in the extract from *Wa*UM3 (negative control) (see **Figure S1**). These results show that yeast strain *Wa*F17.12 from *An. stephensi* [25] releases a protein molecule in the cultivation medium that is labelled by the monoclonal antibody directed against *Wa*KT; this indicates that *Wa*F17.12 likely secretes a killer toxin.

**Figure 1:**
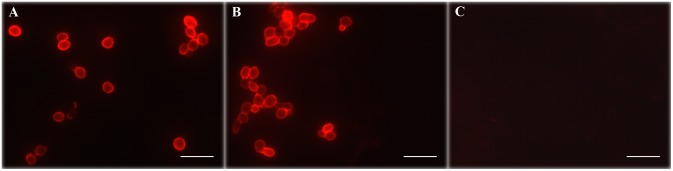
*In vitro* detection of *Wa*KT by IFA assay using mAbKT4. Free yeast cells of *W. anomalus* strains *Wa*F17.12 (A), *Wa*ATCC 96603 (B) and *Wa*UM3 (C) are shown. Red signal shows *Wa*KT concentrated in the yeast cell wall layers of *Wa*F17.12 (A) and *Wa*ATCC 96603 (B) (bars = 20 µm). Phace contrast of panels A, B and C are shown in Figure S6.

Further evidence for the capacity of *Wa*F17.12 to produce a killer toxin was obtained by immunofluorescence assay (IFA) using mAbKT4 *in vitro*, on free yeast cells, and *in vivo*, in the mosquito. Staining with mAbKT4 produced a clear fluorescent signal onto the cell surface of free yeast cells from strains *Wa*F17.12 and *Wa*ATCC 96603, while no signal was revealed in *Wa*UM3 (**Figure 1A, B and C** respectively). Taken together, the results of Western blot analysis and *in vitro* IFA on free cells are coherent, and provide evidence that strain *Wa*F17.12, once isolated from mosquito and cultivated in stimulating conditions, produces *Wa*KT.

Afterwards, IFA assay was carried out *in vivo* in *An. stephensi*, checking the toxin presence in mosquito organs that are known to harbour *W. anomalus* [25]. Staining with mAbKT4 revealed very occasional and faint *Wa*KT signals within the analysed guts and gonads. To investigate if the observed limited staining could derive from a scarce production of the killer toxin in the mosquito body, we carried out an *in vivo* IFA assay using mAbKT4 after feeding mosquitoes with a sugar meal containing yeast cells stimulated *in vitro*. Mosquitoes diet was supplemented for two days with *Wa*F17.12, *Wa*ATCC 96603 (*Wa*KT-producing strain) or *Wa*UM3 (*Wa*KT-non-producing strain), except a control group fed on sterile sugar solution. After each yeast treatments mosquitoes were fed on sterile sugar solution and were then dissected for the recovery of the organs (guts and gonads) at specific time points, for IFA observations. In **table 1** are reported the percentages of positive samples detected on the 10^th^, 20^th^ or 27^th^ day after each yeast treatments (detailed data on male and female organs are given in **table S2**).

**Table 1:** *In vivo* detection of *Wa*KTs in *An. stephensi* by IFA with mAbKT4.

Days after yeast introduction	Mosquito feeding	*Wa*KT detection %^( )°^
10	(i) Sterile sugar solution	**29%** (5*/17)
	(ii) Sugar solution plus *Wa*ATCC	**50%** (8/16)
	(iii) Sugar solution plus *Wa*F17.12	**56%** (9/16)
	(iv) Sugar solution plus *Wa*UM3	**19%** (3*/16)
20	(i) Sterile sugar solution	**60%** (9*/15)
	(ii) Sugar solution plus *Wa*ATCC	**93%** (13/14)
	(iii) Sugar solution plus *Wa*F17.12	**92%** (11/12)
	(iv) Sugar solution plus *Wa*UM3	**50%** (8*/16)
27	(i) Sterile sugar solution	**54%** (7*/13)
	(ii) Sugar solution plus *Wa*ATCC	**83%** (10/12)
	(iii) Sugar solution plus *Wa*F17.12	**92%** (11/12)
	(iv) Sugar solution plus *Wa*UM3	**42%** (5*/12)
	Total samples analysed: 171	

( )° *Wa*KT positive organs/total organs analyzed.

*Occasional *Wa*KT signals were detected in these samples.

**Figure 2:**
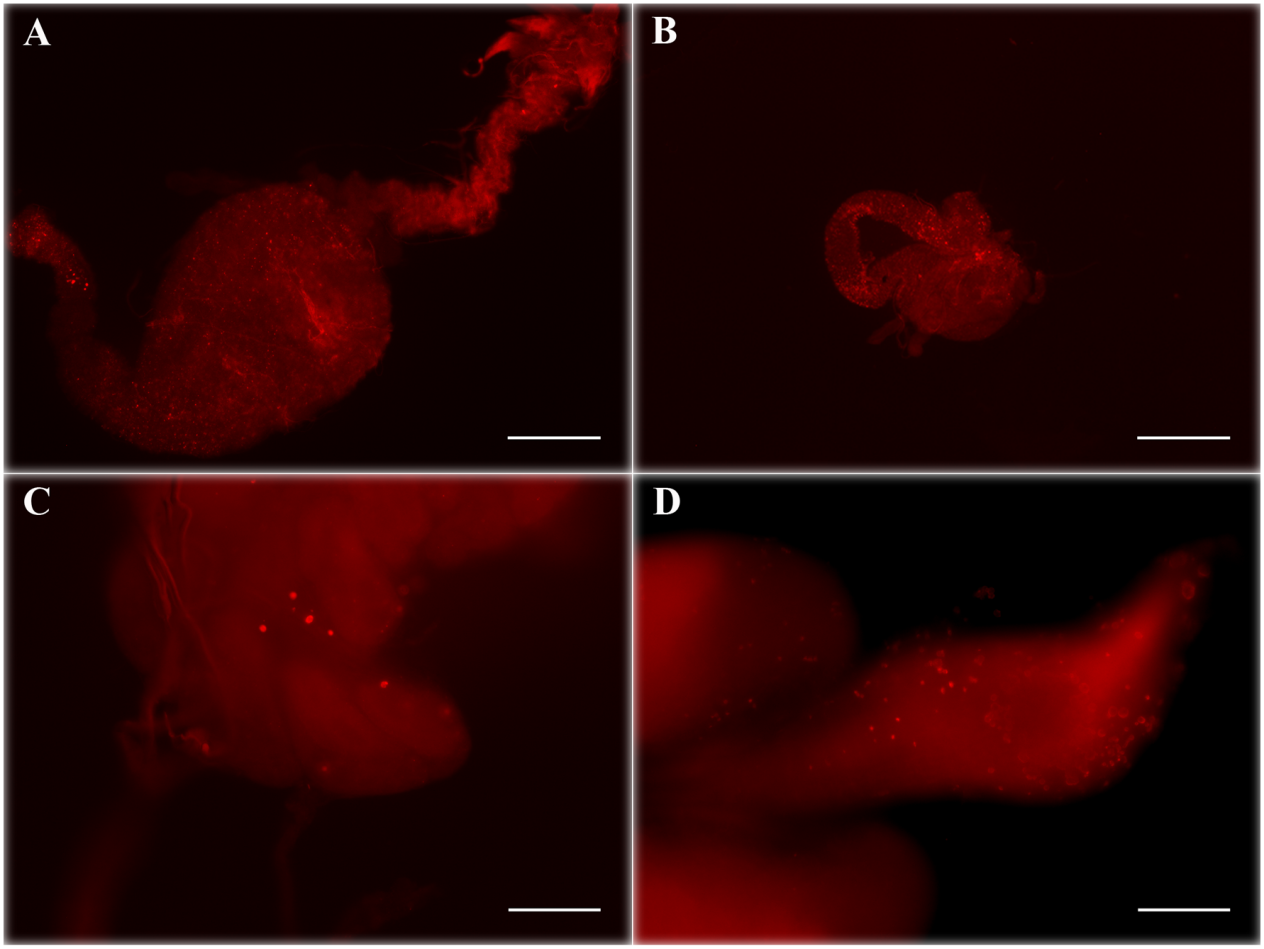
*In vivo* detection of *Wa*F17.12-KT by IFA assay using mAbKT4. Red stained yeasts are visible in the mosquito organs after feeding sugar solution supplemented with *Wa*F17.12 cells cultivated in *Wa*KT stimulating condition: Female (A) and male (B) guts analyzed on the 10^th^ day after yeast introduction (bar = 200 µm), female (C) and male gonads (D) analyzed respectively on the 20^th^ and 27^th^ day after yeast introduction (bar = 50 µm). Negative control (gut treated with secondary antibody only) is shown in Figure S7.

**Figure 3:**
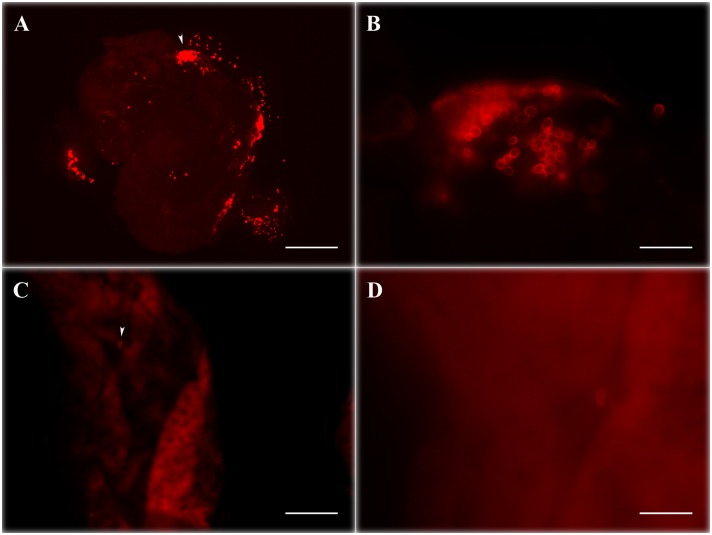
*Wa*F17.12-KT fluorescence signal comparison in female mosquito midguts with or without yeast introduction. Female mosquito midguts on the 10^th^ day after feeding with sugar solution enriched with stimulated *Wa*F17.12 (A and B) and sterile sugar solution (C and D). Red stained yeasts are abundantly visible in (A), while only few cells are detected in the control sample (C). Images B and D (bar = 20 µm) are magnification of samples shown in A and C respectively (bar = 200 µm) White arrow in A and C indicates the section of sample enlarged in B and D.

IFA analysis at different time points highlighted that both *Wa*F17.12 and *Wa*ATCC 96603, once stimulated in culture and reintroduced in the mosquitoes, were characterized by an intense *Wa*KT signal. In the majority of the samples analyzed, *Wa*KT signal was detected up to four weeks after the introduction of the yeast-enriched meal (**Table 1**). After *Wa*F17.12 introduction, fluorescence analysis revealed the presence of an abundant *Wa*KT signal in both female and male midguts (**Figure 2A and B**; see also immunolocalization in **Figure S2**) and gonads (**Figure 2C and D**). Similar staining was observed in the samples fed with the reference strain *Wa*ATCC 96603 (not shown). In contrast, in mosquitoes supplemented with *Wa*UM3 and in the control group, the percentages of positive samples are lower than in the other mosquitoes and we observed only a few faintly stained yeast cells, that might perhaps represent resident (not introduced) yeasts. **Figure 3** shows a comparison of the typical *Wa*KT signal detected in mosquitoes fed with *Wa*F17.12 or with sterile sugar solution only.

IFA analysis also revealed some differences between guts and gonads: we observed that the *Wa*KT signal on the 10^th^ day was detected almost exclusively in the guts, whereas in the following two time points the number of positive gonads increased and became comparable to that of the guts (**Table S2**).

Significant *Wa*KT signal was detected even in part of the offspring of *Wa*F17.12 fed mosquitoes, suggesting a vertical transmission of the yeasts and a long-term effect of the *in vitro* induction of KT secretion (**Figure S3**).

### Crude WaF17.12-KT Activity Assays

Experiments performed using yeast culture supernatants showed antimicrobial activity of *Wa*F17.12 against the susceptible strain *Sc*ATCC 2601 [34], belonging to the species *S. cerevisiae*. Specifically the growth of the target yeast *Sc*ATCC 2601 was inhibited when inoculated in the liquid culture medium obtained from the supernatants of *Wa*ATCC 96603 (*Wa*KT-producing strain) and *Wa*F17.12 grown at condition that stimulate *Wa*KT production, but not using supernatant from *Wa*UM3 (*Wa*KT-non-producing strain) (see **Figure S4**).

**Figure 4:**
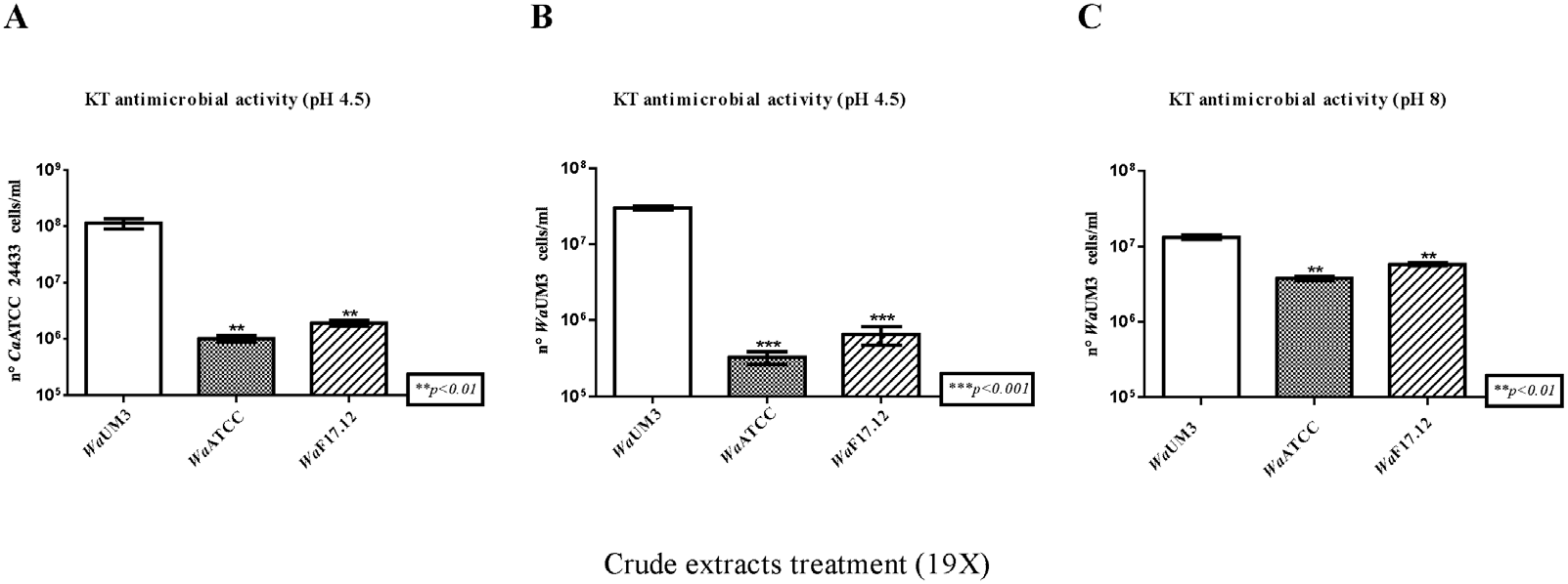
Crude *Wa* F17.12-KT extract activity assays. The susceptible strains *Ca*ATCC 24433 (A) and *Wa*UM3 (B and C) were incubated for 12 h at 26°C at pH 4.5 (A and B) and 8 (C) with *Wa*KT crude extracts (19X) from *Wa*F17.12, *Wa*ATCC 96603 (*Wa*KT-producing strain) or *Wa*UM3 (*Wa*KT non-producing strain). Crude *Wa*KT activity was evaluated using the trypan blue test reporting the number of vital cells/ml of the two target strains and the values are reported as mean±SEM (n = 6). Asterisks refers to the statistical significance of the differences, according to the One Way ANOVA and Bonferroni’s Multiple Comparation Tests.

In-depth antimicrobial activity assays were carried out using crude *Wa*KT extracts from *Wa*F17.12, *Wa*ATCC 96603 and *Wa*UM3 against two additional *Wa*KT susceptible yeast strains, *Ca*ATCC 24433 [32], belonging to the species *C. albicans*, and *Wa*UM3 itself [30], [31]. The results obtained using different concentrations of the crude extracts (50X and 19X) were comparable and data about the lower concentration assay are shown in **figure 4** (those from the 50X concentration are not shown). Since it is reported that *Wa*KT activity shows its maximum around acidic pH values, with a peak at pH 4.5 [21], [33], this was the pH value used in assays for both sensitive target strains (**Figure 4A and B**). To test possible killer activity at not ideal pH conditions, we performed an additional assay at pH 8 (**Figure 4C**) on *Wa*UM3, considering this target as the most suitable one [4]. In the three assays the results of toxin activity revealed a growth inhibition of both susceptible strains when treated with *Wa*KT crude extracts from *Wa*F17.12 and *Wa*ATCC 96603, while no effect was detected with *Wa*UM3 extract. More in detail, cell counts of *Ca*ATCC 24433 (**Figure 4A**) and *Wa*UM3 (**Figure 4B and C**) showed a growth inhibition after treatment with both of the crude extracts from the two *Wa*KT-producing strains (p<0.01, p<0.001 and p<0.01 respectively). These data confirmed that the susceptibility of the two target strains to toxic activity of *Wa*F17.12 was comparable to that exerted by the reference strain *Wa*ATCC 96603. Importantly, significant antimicrobial activity against *Wa*UM3 was observed also at pH 8 (**Figure 4C**) although the toxic effect appeared to be lower than those at acidic pH, in agreement with published results [33]. Bonferroni’s tests have been performed comparing the number of cells/ml of the target strains after each treatment with crude extracts from *Wa*F17.12, or *Wa*ATCC 96603 (positive control), versus treatment with crude extract from *Wa*UM3 (negative control).

Since a *Wa*KT-mediated activity on Gram-negative bacteria has been described [9], we investigated the effect of a culture *Wa*F17.12 stimulated for KT production on the *An. stephensi* bacterial symbiont *Asaia* sp., that is known to massively inhabit the mosquito midgut and gonads [27]. The assays showed that the growth of *Asaia* sp. colonies *in vitro* was not inhibited by the *Wa*F17.12, suggesting that this yeast does not exert its antimicrobial activity against *Asaia* symbionts (see **Figure S5**).

### Sequence Analysis of WaF17.12 Exoglucanase-encoding Genes EXG1 and EXG2

The *EXG1* and *EXG2* complete coding genes were amplified, cloned and sequenced. We identified two Open Reading Frame (ORF) sequences of 1497 bps and 1284 bps, respectively. Corresponding amino acid sequences are of 499 residues in the case of *EXG1* and 428 for *EXG2*.

The *EXG1* and *EXG2* sequences showed a 100% and 99% similarity respectively with the GenBank *W. anomalus* sequences JQ734563 and JQ734566 [20]. Sequences of *Wa*F17.12 *EXG1* and *EXG2* genes were deposited through the EMBL-Bank under the accession numbers HG316785 (*EXG1*) and HG316786 (*EXG2*).

## Discussion

### The Yeast Strain WaF17.12 Isolated from Mosquito Secretes WaKT Both *In vitro* and *In vivo*

Western blot analysis of cultural crude extracts showed that strain *Wa*F17.12, isolated from the malaria vector *An. stephensi*, releases a molecule in the medium that is stained by mAbKT4, a monoclonal antibody targeted on killer yeast toxins. By *in vitro* IFA assay on free cells, we verified that cultivated *Wa*F17.12 yeasts also produce a molecule recognized by mAbKT4, concentrated at the surface of the yeast. This is in accordance with published results indicating toxin secretion through the cell wall in *W. anomalus Wa*KT-producing strains [36], [37]. Overall, the above results strongly suggest that *Wa*F17.12 yeast strain from mosquitoes produces a killer toxin.

IFA analyses also pinpointed that when cultures of *Wa*F17.12 stimulated for toxin production were reintroduced within the mosquito by the diet, the *Wa*KT signal was still present several weeks after the yeast reintroduction, i.e. for the entire mosquito life span (a relevant mAbKT4-staining was found even in the offspring of these mosquitoes). Particularly, the toxin signal was detected in the midgut and gonads, in both male and female mosquitoes. The *Wa*KT signal in mosquitoes fed with *Wa*F17.12 (or the reference strain *Wa*ATCC 96603) was quite abundant especially within the female midgut. Instead, the mosquitoes fed with sterile sugar solution or with *Wa*UM3 showed few stained yeast cells. Reasonably, this occasional *Wa*KT signal could derive from the presence of residing (not introduced) yeasts capable of producing the toxin (and possibly related with *Wa*F17.12).

### Crude WaKT Extract from WaF17.12 shows *In vitro* Antimicrobial Activity

Bio-assays demonstrated that *Wa*F17.12 exhibits antimicrobial effects against different susceptible strains, inhibiting their growth in cultivation media. Similarly to other strains of *W. anomalus* that have been described to be active against *C. albicans*, we demonstrated that *Wa*F17.12 exerted an antimicrobial effect *in vitro* on a strain from this species, *Ca*ATCC 24433. Since ascomycetous yeasts are frequently described to infect mosquitoes [24], [38], [39], future studies could investigate the possible detrimental effects of killer yeast strains against other yeasts sharing the same habitat within the insect body.

*Wa*KT activity assays at pH 4.5 and pH 8 demonstrated that the killer activity of *Wa*F17.12 against *Wa*UM3 spans a wide range of pH values. This finding fits well with possible antimicrobial competition of *W. anomalus* in the mosquito body, since different physiological conditions can occur *in vivo*, for example in the different organs as well as at the intestinal level depending on the diet. Particularly, it is described that pH in the female midgut can vary around basic values after a blood meal [40], [41]. Concerning temperature, our experiments were carried out at 26°C, the suggested condition for toxic assays [32]. Nevertheless, antimicrobial tests demonstrated that the maximum *Wa*KTs activity ranges from 18°C to 30°C and its decrement starts at around 40°C [33]. Adaptability of KT activity to different pH values and temperatures is an important requisite for potential applications of killer yeast strains in the biotechnological control of mosquito transmitted diseases. In this context, the inefficacy of *Wa*F17.12-KT against *Asaia* sp. supports a feasible cohabitation of the bacteria and the yeast in the mosquito midgut and gonads, suggesting the possible synergistic use of *W. anomalus* in combination with other microbial candidates for SC.

### Exoglucanase-encoding Genes as Possible Mediators of W. anomalus Killer Activity

Antimicrobial activity of toxins produced by killer yeasts is well documented in literature [21], [42], [43], while little is known about the genetics of this antimicrobial phenotype. Some indications suggested that *Wa*KTs are chromosomally inherited [44], but the chromosomal localization of the genes is still missing [5]. Of particular interest was the identification of genes responsible for the synthesis of exo- β-1,3-glucanase [19] and the characterization of its enzymatic activity, adapted to a wide range of temperature and acidic pH [45]–[47]. This activity is responsible for the cleavage of the β-glycosidic bond of β-glucan, a major cell wall component of yeasts, [33]. Here, we reported the identification of two genes in the mosquito yeast strain *Wa*F17.12, *EXG1* and *EXG2*, that encode for exo-1,3-*β*-glucanases. Nevertheless hypothesis of a possible association between antimicrobial phenotype of *Wa*F17.12with exo-β-1,3-glucanase activity needs further characterization of these genes. Particularly, the amplification of both *EXG*-genes also in the non-killer strain *Wa*UM3 lays the basis for later studies encompassing possible polymorphisms and differences in the transcriptional activity of these genes in KT-producing and non KT-producing strains. A clarification of this aspect may contribute to explain the molecular basis of the killer phenomenon.

### Conclusions and Prospects for Future Applications

The results here reported show that mosquito yeast strain *Wa*F17.12: (i) secretes a molecule recognized by a monoclonal antibody specific for killer yeast toxins; (ii) possesses antimicrobial activity against sensitive yeast strains/species. This allows us to conclude that the mosquito yeast *Wa*F17.12 can be regarded as a killer yeast, capable of producing a killer toxin. We would thus indicate this toxin as *Wa*F17.12-KT.

*W. anomalus* yeasts had been detected in mosquitoes a few years ago, but their biological role in these insects is still unknown [24], [25]. Arthropod-associated *W. anomalus* strains could exert a nutritional role [22] and/or a protective function mediated by antimicrobial activity against pathogens [48], and this led us to hypothesize that *W. anomalus* might play similar roles also in mosquitoes [24], [25]. The novel finding that the strain isolated from *An. stephensi* displays an antimicrobial phenotype supports the hypothesis of a protective function. The protective role of symbiotic microorganisms against pathogens in different arthropod species is emerging as a promising research field, with a high potential for future applications toward the control of vector-borne diseases [29], [49]. However, most of the papers so far published in this field deal with bacterial symbionts, and the work here presented provides the first evidence for the presence of a yeast expressing a killer phenotype in mosquitoes.

The presence of a *W. anomalus* killer strain in the female mosquito midgut is quite interesting, considering that the gut represents a numeric bottleneck during the life cycle of *Plasmodium*, and this could make the parasites more vulnerable to the action of antagonistic microorganisms [50]. Future studies should thus investigate whether KT-producing strains of *W. anomalus* possess anti-*Plasmodium* activity, and whether enough toxin is produced by the yeasts naturally present in the mosquito gut. On the other hand, our results show that cultivated yeasts can easily colonize mosquitoes, and then continue with toxin production for at least four weeks. Killer yeasts could also be selected for an increased secretion of KT at conditions that simulates the mosquito gut, and for more effective anti-malaria activity. A possible association between antimicrobial phenotype of KT-producing strains with exo-β-1,3-glucanase seems very interesting, particularly considering that the enzyme target carbohydrate (β-1,3-glucan) is already known to be present in some parasites oocyst walls as *Toxoplasma* and *Eimeria* [51] and implicated in the immunoprotection against *Plasmodium berghei* infection in mice [52].

Yeasts naturally effective against malaria (or even after selection for increased activity) would likely be more easy to be accepted by the public opinion for field applications, compared to the genetically modified microorganisms foreseen by the so-called paratransgenesis approach [53].

## Data Accessibility

DNA sequences are accessible at http://www.ebi.ac.uk/ena/data/view/HG316785-HG316786.

## Supporting Information

Figure S1 **Western blot analysis of** ***Wa*** **KT crude extracts.** *Wa*F17.12, *Wa*ATCC 96603 (*Wa*KT-producer strain) and *Wa*UM3 (*Wa*KT-not-producer strain) crude extracts, obtained after concentration of supernatants from yeast cultures stimulated for *Wa*KT secretion, were analyzed using mAbKT4. (1) Standard molecular weight marker expressed in kDa and *Wa*KT crude extracts from (2) *Wa*UM3, (3) *Wa*ATCC 96603 and (4) *Wa*F17.12. A single band at around 250 kDa was revealed in extracts from *Wa*ATCC 96603 and *Wa*F17.12.
(DOC)

Figure S2 **Immunolocalization of** ***Wa*** **F17.12-KT in** ***An. Stephensi*** **abdominal sections.** Abdominal histological sections of adult female mosquitoes treated with sterile sugar solution (A) or with sugar solution enriched with stimulated yeast cultures of *Wa*F17.12 (B). White asterisk and black arrow in (B) indicate positive staining surrounding the intestinal epithelium and the presence of *Wa*F17.12 cells in the midgut lumen (ML), respectively. *Wa*F17.12-KT signal in panel (B) is shown in the mosquito gut on the 10^th^ day post yeast treatment. mAbKT4 is able to recognize both the *Wa*F17.12-KT on the surface of the yeast cell and in soluble form, as demonstrated by IFA and Western blot analysis, respectively. Interestingly, the staining obtained by immunohistochemistry appears to localize on both yeast cells surface and intestinal epithelium, supporting the hypothesis that *Wa*F17.12-KT is secreted in vivo and present in the gut lumen.
(DOC)

Figure S3 ***In vivo*** **detection of** ***Wa*** **F17.12-KT in mosquito offspring by IFA assay using mAbKT4.** Red stained yeasts are visible (white arrow) in the female mosquito midgut (F_1_ generation from parental mosquitoes fed for two days with sugar solution plus stimulated *Wa*F17.12 cells) (bar = 50 µm).
(DOC)

Figure S4 **Antimicrobial activity of** ***Wa*** **F17.12 against** ***S. cerevisiae*** **.** The susceptible strain *Sc*ATCC 2601 was inoculated in fresh YPD medium (A) or in filtered broth culture surnatants of the strains *Wa*UM3 (B), *Wa*ATCC 96603 (C) and *Wa*F17.12 (D) grown at conditions that stimulate *Wa*KT production. After an overnight incubation at 26°C and 70 rpm, optical densities (OD) of each *Sc*ATCC 2601 culture were measured. *Sc*ATCC 2601 growth rates compared to growth control (A) showed to be slightly affected in (B), whereas there is a strong decrease in (C) and (D). (A): 5.97 OD (2×10^8^ cells/ml*); (B): 3.83 OD (1.3×10^8^ cells/ml*); (C): 1.04 OD (3.5×10^7^ cells/ml*); (D): 1.04 OD (3.5×10^7^ cells/ml*). *Concentration (cells/ml) of *Sc*ATCC 2601 has been evaluated by the assessment of a standard growth curve.
(DOC)

Figure S5 **Growth inhibition evaluation of** ***Wa*** **F17.12-KT against** ***S. cerevisiae*** **and** ***Asaia*** **sp.** Cultures of *Sc*ATCC 2601 (A) and *Asaia* sp. (B) were seeded on YPD agar and GLY agar, respectively. The plates were incubated for 72 h at 20°C with three dilution of the activated *Wa*F17.12 culture: 10^9^ cells/ml (1 and 4), 10^8^ cells/ml (2 and 5) and 10^7^ cells/ml (3 and 6). *Wa*UM3 culture (10^9^ cell/ml) was spotted as negative control (N). A growth inhibition halo is distinguishable surrounding the yeast colonies only in the plate A (black arrow). The presence of a pink ring of *Asaia* sp. around the yeast colonies (black arrow) can be observed in plate B.
(DOC)

Figure S6 ***In vitro*** **detection of** ***Wa*** **KT by IFA assay using mAbKT4 (phase contrast of** **Fig. 1** **in the main test).** Free yeast cells of *W. anomalus* strains *Wa*F17.12 (A), *Wa*ATCC 96603 (B) and *Wa*UM3 (C) phase contrast images corresponding to images of Fig. 1 (A), (B) and (C) respectively.
(DOC)

Figure S7 ***In vivo*** **detection of** ***Wa*** **F17.12-KT by IFA assay using mAbKT4 (negative control for IFA experiments).** Female gut from mosquito fed with cultivated *Wa*F17.12 after treatment with secondary antibody only.
(DOC)

Table S1 **Oligonucleotides used in** ***EXG1*** **and** ***EXG2*** **genes molecular analysis.** List of primers used in *EXG* genes sequence analysis.
(DOC)

Table S2 **Detection of** ***Wa*** **KT signal in mosquito organs by IFA with mAbKT4.** Four cages containing mosquitoes fed with: (i) Sterile sugar solution, (ii) sugar solution enriched with *Wa*ATCCC 96603 (*Wa*KT-producing strain), (iii) sugar solution enriched with *Wa*F17.12 and (iv) sugar solution enriched with *Wa*UM3 (*Wa*KT non-producing strain). Organs were dissected 10, 20 and 27 days after yeast introduction and analyzed using a fluorescence microscope.
(DOC)
