# Supplementary material for: A Wickerhamomyces anomalus Killer Strain in the Malaria Vector Anopheles stephensi
Source: PLoS One. 2014 May 1;9(5):e95988. doi: 10.1371/journal.pone.0095988 (PMC4006841; doi:10.1371/journal.pone.0095988)
Supplement: Figure S1 — Western blot analysis of Wa KT crude extracts. WaF17.12, WaATCC 96603 (WaKT-producer strain) and WaUM3 (WaKT-not-producer strain) crude extracts, obtained after concentration of supernatants from yeast cultures stimulated for WaKT secretion, were analyzed using mAbKT4. (1) Standard molecular weight marker expressed in kDa and WaKT crude extracts from (2) WaUM3, (3) WaATCC 96603 and (4) WaF17.12. A single band at around 250 kDa was revealed in extracts from WaATCC 96603 and WaF17.12. (DOC) [file pone.0095988.s001.doc]

**SUPPORTING INFORMATION**

**Figure S1. Western blot analysis of *Wa*KT crude extracts.**

*Wa*F17.12, *Wa*ATCC 96603 (*Wa*KT-producer strain) and *Wa*UM3 (*Wa*KT-not-producer strain) crude extracts, obtained after concentration of supernatants from yeast cultures stimulated for *Wa*KT secretion, were analyzed using mAbKT4. (1) Standard molecular weight marker expressed in kDa and *Wa*KT crude extracts from (2) *Wa*UM3, (3) *Wa*ATCC 96603 and (4) *Wa*F17.12. A single band at around 250 kDa was revealed in extracts from *Wa*ATCC 96603 and *Wa*F17.12.

**
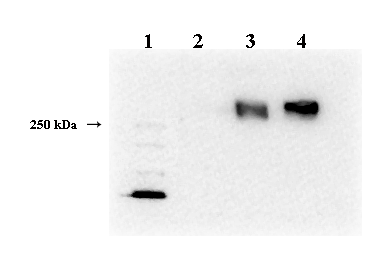
**
