# Supplementary material for: A Wickerhamomyces anomalus Killer Strain in the Malaria Vector Anopheles stephensi
Source: PLoS One. 2014 May 1;9(5):e95988. doi: 10.1371/journal.pone.0095988 (PMC4006841; doi:10.1371/journal.pone.0095988)
Supplement: Figure S2 — Immunolocalization of Wa F17.12-KT in An. Stephensi abdominal sections. Abdominal histological sections of adult female mosquitoes treated with sterile sugar solution (A) or with sugar solution enriched with stimulated yeast cultures of WaF17.12 (B). White asterisk and black arrow in (B) indicate positive staining surrounding the intestinal epithelium and the presence of WaF17.12 cells in the midgut lumen (ML), respectively. WaF17.12-KT signal in panel (B) is shown in the mosquito gut on the 10th day post yeast treatment. mAbKT4 is able to recognize both the WaF17.12-KT on the surface of the yeast cell and in soluble form, as demonstrated by IFA and Western blot analysis, respectively. Interestingly, the staining obtained by immunohistochemistry appears to localize on both yeast cells surface and intestinal epithelium, supporting the hypothesis that WaF17.12-KT is secreted in vivo and present in the gut lumen. (DOC) [file pone.0095988.s002.doc]

**SUPPORTING INFORMATION**

**Figure S2. Immunolocalization of *Wa*F17.12-KT in *An. stephensi* using mAbKT4.**

Abdominal histological sections of adult female mosquitoes treated with sterile sugar solution (A) or with sugar solution enriched with stimulated yeast cultures of *Wa*F17.12 (B). White asterisk and black arrow in (B) indicate positive staining surrounding the intestinal epithelium and the presence of *Wa*F17.12 cells in the midgut lumen (ML), respectively. *Wa*F17.12-KT signal in panel (B) is shown in the mosquito gut on the 10^th^ day post yeast treatment.


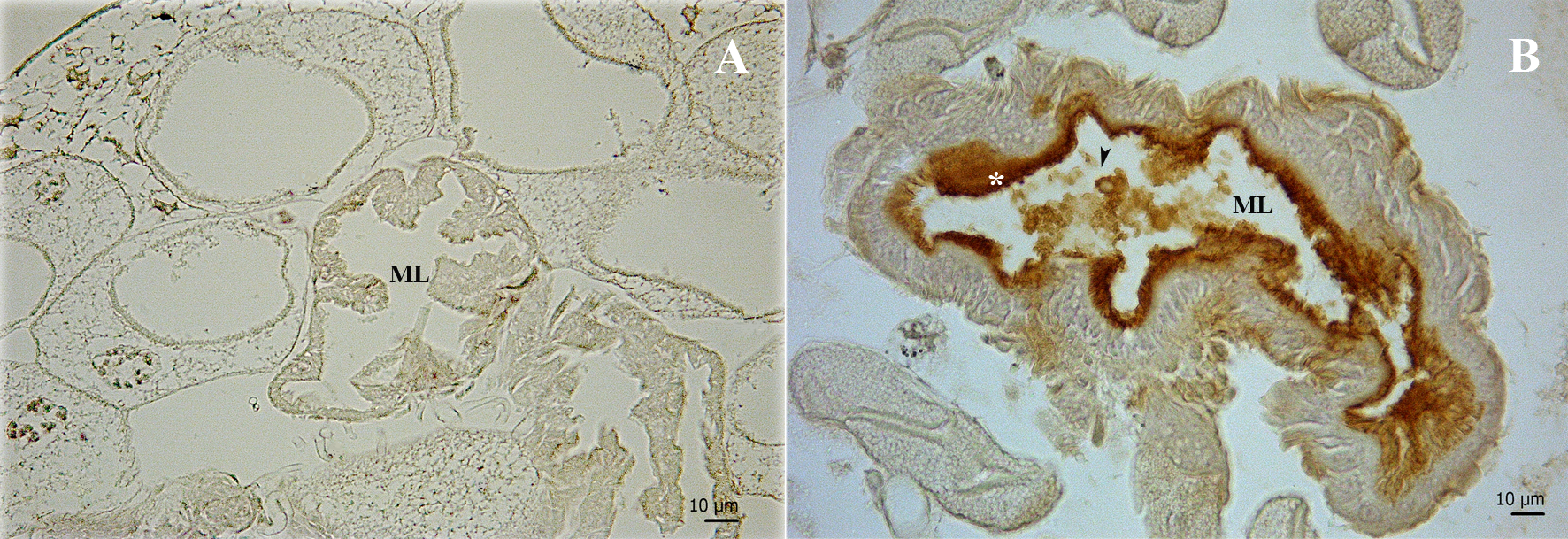


mAbKT4 is able to recognize both the WaF17.12-KT on the surface of the yeast cell and in soluble form, as demonstrated by IFA and Western blot analysis, respectively. Interestingly, the staining obtained by immunohistochemistry appears to localize on both yeast cells surface and intestinal epithelium, supporting the hypothesis that WaF17.12-KT is secreted in vivo and present in the gut lumen.
