# Supplementary material for: A Wickerhamomyces anomalus Killer Strain in the Malaria Vector Anopheles stephensi
Source: PLoS One. 2014 May 1;9(5):e95988. doi: 10.1371/journal.pone.0095988 (PMC4006841; doi:10.1371/journal.pone.0095988)

**SUPPORTING INFORMATION**

**Figure S3. *In vivo* detection of *Wa*F17.12-KT in mosquito offspring by IFA assay using mAbKT4.**

Red stained yeasts are visible (white arrow) in the female mosquito midgut (F_1_ generation from parental mosquitoes fed for two days with sugar solution plus stimulated *Wa*F17.12 cells) (bar= 50µm).


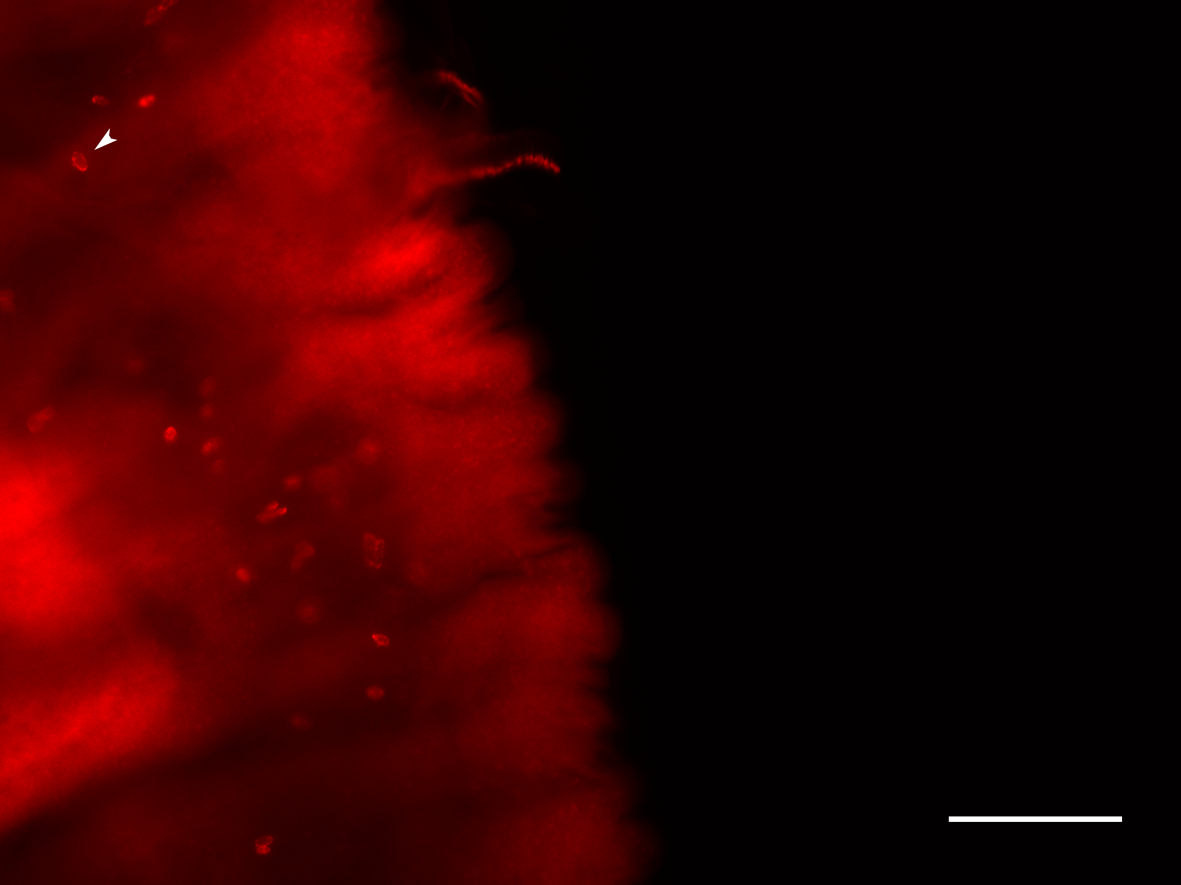

Supplement: Figure S3 — In vivo detection of Wa F17.12-KT in mosquito offspring by IFA assay using mAbKT4. Red stained yeasts are visible (white arrow) in the female mosquito midgut (F1 generation from parental mosquitoes fed for two days with sugar solution plus stimulated WaF17.12 cells) (bar = 50 µm). (DOC) [file pone.0095988.s003.doc]
