# Supplementary material for: A Wickerhamomyces anomalus Killer Strain in the Malaria Vector Anopheles stephensi
Source: PLoS One. 2014 May 1;9(5):e95988. doi: 10.1371/journal.pone.0095988 (PMC4006841; doi:10.1371/journal.pone.0095988)
Supplement: Figure S4 — Antimicrobial activity of Wa F17.12 against S. cerevisiae . The susceptible strain ScATCC 2601 was inoculated in fresh YPD medium (A) or in filtered broth culture surnatants of the strains WaUM3 (B), WaATCC 96603 (C) and WaF17.12 (D) grown at conditions that stimulate WaKT production. After an overnight incubation at 26°C and 70 rpm, optical densities (OD) of each ScATCC 2601 culture were measured. ScATCC 2601 growth rates compared to growth control (A) showed to be slightly affected in (B), whereas there is a strong decrease in (C) and (D). (A): 5.97 OD (2×10^8^ cells/ml*); (B): 3.83 OD (1.3×10^8^ cells/ml*); (C): 1.04 OD (3.5×10^7^ cells/ml*); (D): 1.04 OD (3.5×10^7^ cells/ml*). *Concentration (cells/ml) of ScATCC 2601 has been evaluated by the assessment of a standard growth curve. (DOC) [file pone.0095988.s004.doc]

**SUPPORTING INFORMATION**

**Figure S4. Antimicrobial activity of *Wa*F17.12-KT against *S. cerevisiae*.**

The susceptible strain *Sc*ATCC 2601 was inoculated in fresh YPD medium (A) or in filtered broth culture surnatants of the strains *Wa*UM3 (B), *Wa*ATCC 96603 (C) and *Wa*F17.12 (D) grown at conditions that stimulate *Wa*KT production. After an overnight incubation at 26°C and 70 rpm, optical densities (OD) of each *Sc*ATCC 2601 culture were measured. *Sc*ATCC 2601 growth rates compared to growth control (A) showed to be slightly affected in (B), whereas there is a strong decrease in (C) and (D).


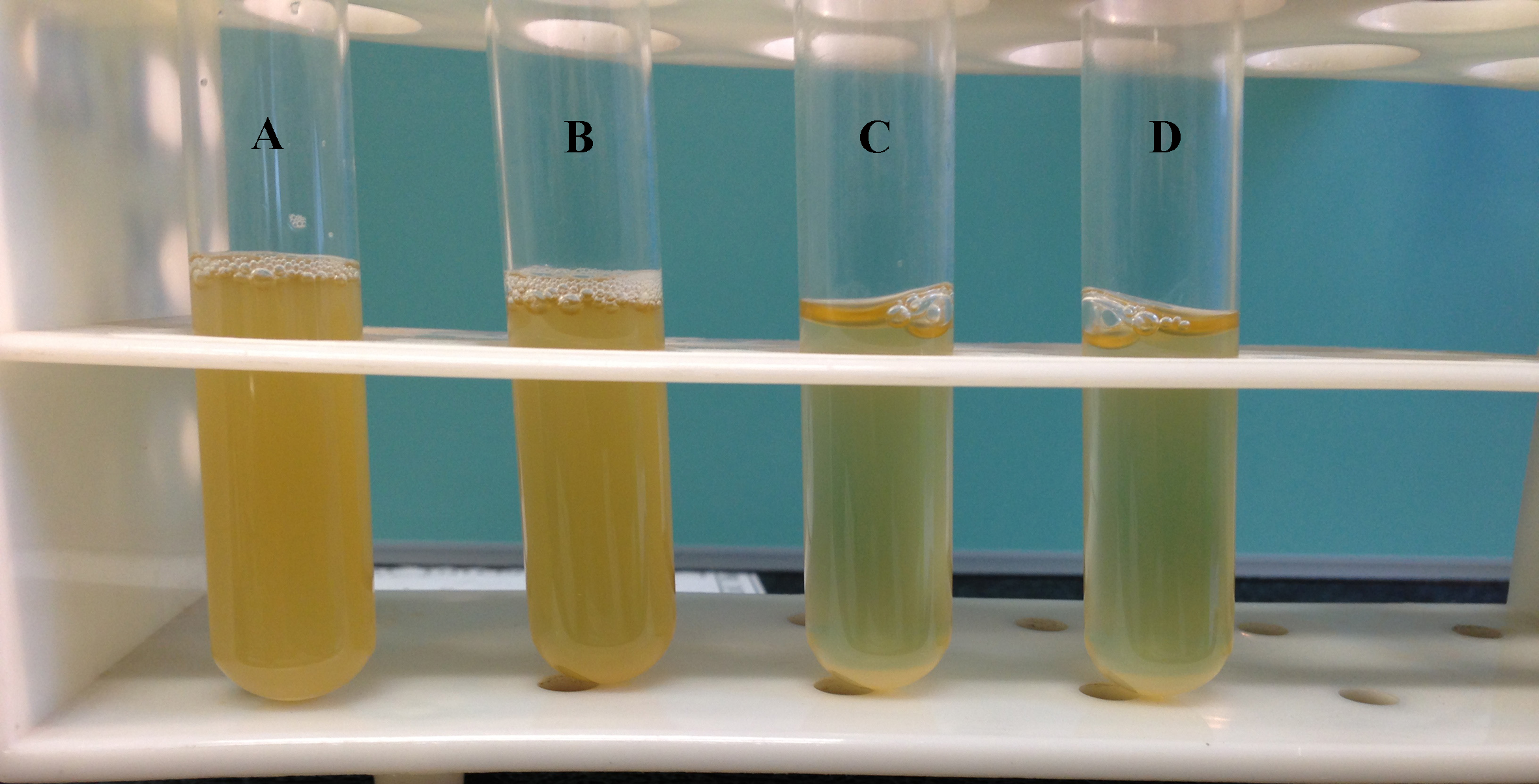


(A): 5.97 OD (2x10^8^ cells/ml*);

(B): 3.83 OD (1.3x10^8^ cells/ml*);

(C): 1.04 OD (3.5x10^7^ cells/ml*);

(D): 1.04 OD (3.5x10^7^ cells/ml*).

*Concentration (cells/ml) of *Sc*ATCC 2601 has been evaluated by the assessment of a standard growth curve.
