# Supplementary material for: A Wickerhamomyces anomalus Killer Strain in the Malaria Vector Anopheles stephensi
Source: PLoS One. 2014 May 1;9(5):e95988. doi: 10.1371/journal.pone.0095988 (PMC4006841; doi:10.1371/journal.pone.0095988)

**SUPPORTING INFORMATION**

**Figure S5. Growth inhibition evaluation of *Wa*F17.12-KT against *Sc*ATCC 2601 and *Asaia* sp.**

Cultures of *Sc*ATCC 2601 (A) and *Asaia* sp. (B) were seeded on YPD agar and GLY agar, respectively. The plates were incubated for 72 h at 20°C with three dilution of the activated *Wa*F17.12 culture: 10^9^ cells/ml (1 and 4), 10^8^ cells/ml (2 and 5) and 10^7^ cells/ml (3 and 6). *Wa*UM3 culture (10^9^ cell/ml) was spotted as negative control (N). A growth inhibition halo is distinguishable surrounding the yeast colonies only in the plate A (black arrow). The presence of a pink ring of *Asaia* sp. around the yeast colonies (black arrow) can be observed in plate B.


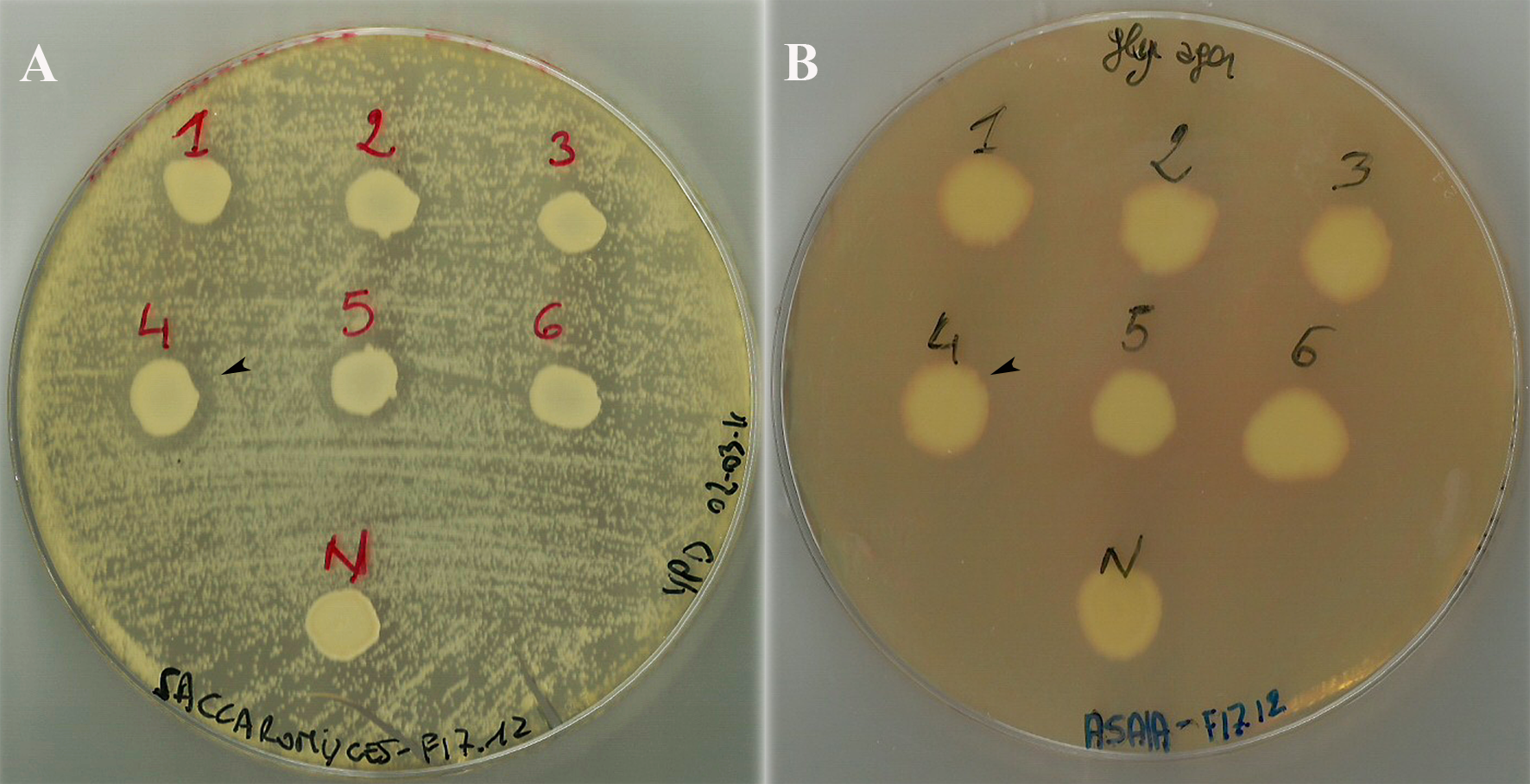

Supplement: Figure S5 — Growth inhibition evaluation of Wa F17.12-KT against S. cerevisiae and Asaia sp. Cultures of ScATCC 2601 (A) and Asaia sp. (B) were seeded on YPD agar and GLY agar, respectively. The plates were incubated for 72 h at 20°C with three dilution of the activated WaF17.12 culture: 109 cells/ml (1 and 4), 108 cells/ml (2 and 5) and 107 cells/ml (3 and 6). WaUM3 culture (109 cell/ml) was spotted as negative control (N). A growth inhibition halo is distinguishable surrounding the yeast colonies only in the plate A (black arrow). The presence of a pink ring of Asaia sp. around the yeast colonies (black arrow) can be observed in plate B. (DOC) [file pone.0095988.s005.doc]
