# Supplementary material for: A Wickerhamomyces anomalus Killer Strain in the Malaria Vector Anopheles stephensi
Source: PLoS One. 2014 May 1;9(5):e95988. doi: 10.1371/journal.pone.0095988 (PMC4006841; doi:10.1371/journal.pone.0095988)
Supplement: Figure S6 — In vitro detection of Wa KT by IFA assay using mAbKT4 (phase contrast of Fig. 1 in the main test). Free yeast cells of W. anomalus strains WaF17.12 (A), WaATCC 96603 (B) and WaUM3 (C) phase contrast images corresponding to images of Fig. 1 (A), (B) and (C) respectively. (DOC) [file pone.0095988.s006.doc]

**SUPPORTING INFORMATION**

**Figure S6. *In vitro* detection of *Wa*KT by IFA assay using mAbKT4 (phase contrast of Fig.1 in the main test).**

Free yeast cells of *W. anomalus* strains *Wa*F17.12 (A), *Wa*ATCC 96603 (B) and *Wa*UM3 (C) phase contrast images corresponding to images of Fig.1 (A), (B) and (C) respectively.

**
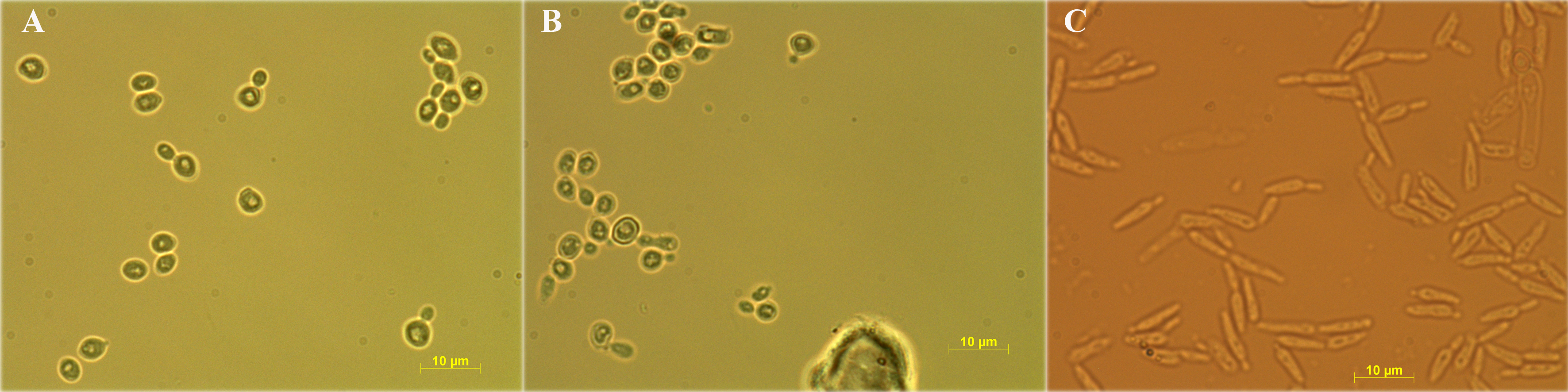
**
