# Supplementary material for: A Wickerhamomyces anomalus Killer Strain in the Malaria Vector Anopheles stephensi
Source: PLoS One. 2014 May 1;9(5):e95988. doi: 10.1371/journal.pone.0095988 (PMC4006841; doi:10.1371/journal.pone.0095988)
Supplement: Figure S7 — In vivo detection of Wa F17.12-KT by IFA assay using mAbKT4 (negative control for IFA experiments). Female gut from mosquito fed with cultivated WaF17.12 after treatment with secondary antibody only. (DOC) [file pone.0095988.s007.doc]

**SUPPORTING INFORMATION**

**Figure S7. *In vivo* detection of *Wa*F17.12-KT by IFA assay using mAbKT4 (negative control for IFA experiments).**

Female gut from mosquito fed with cultivated *Wa*F17.12 after treatment with secondary antibody only.

**
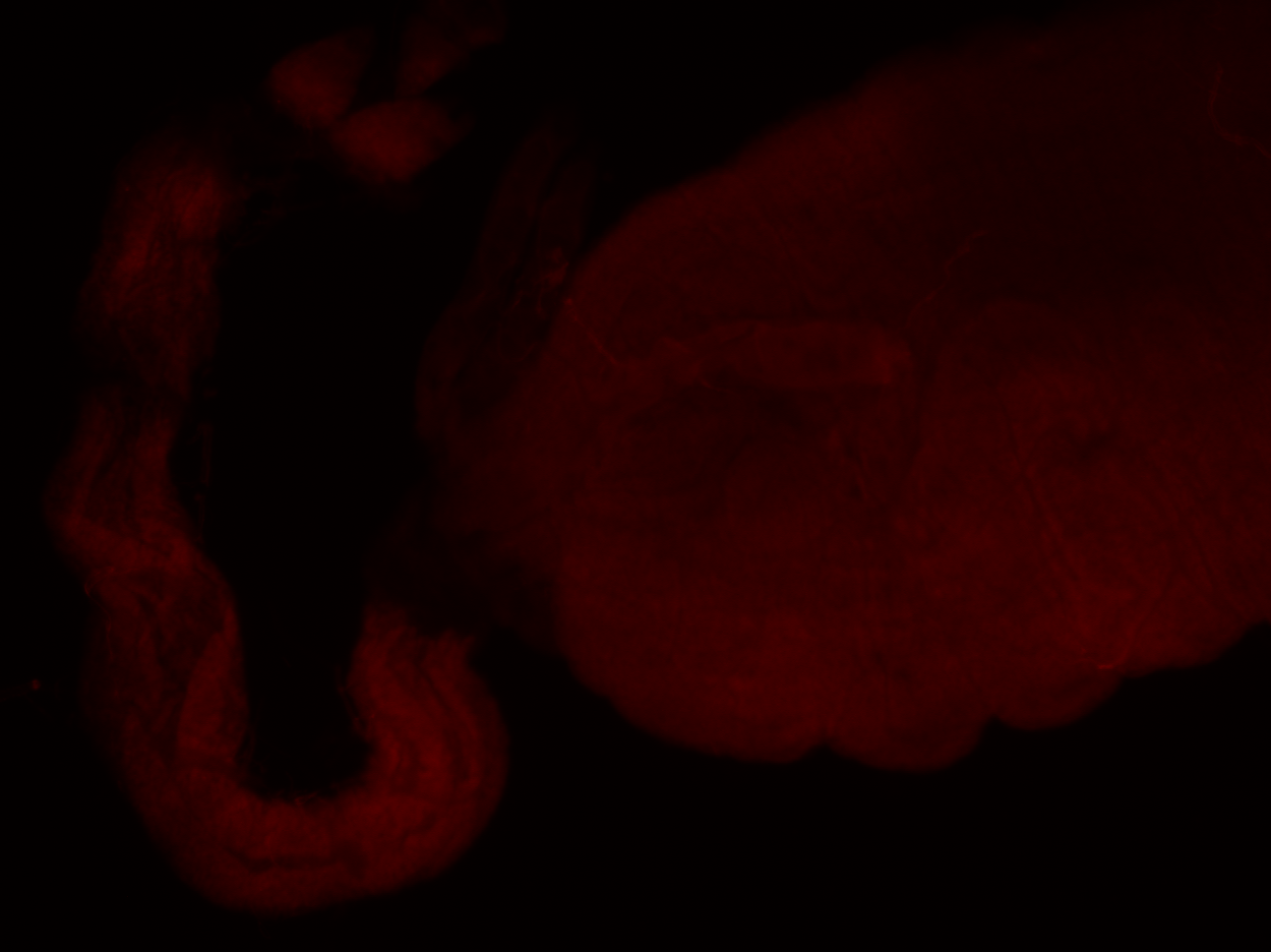
**
