# Supplementary material for: A Wickerhamomyces anomalus Killer Strain in the Malaria Vector Anopheles stephensi
Source: PLoS One. 2014 May 1;9(5):e95988. doi: 10.1371/journal.pone.0095988 (PMC4006841; doi:10.1371/journal.pone.0095988)
Supplement: Table S1 — Oligonucleotides used in EXG1 and EXG2 genes molecular analysis. List of primers used in EXG genes sequence analysis. (DOC) [file pone.0095988.s008.doc]

**SUPPORTING INFORMATION**

**Table S1. Oligonucleotides used in *EXG1* and*EXG2* genes molecular analysis**.

| Oligonucleotides | 5’🡪3’ sequence |
| --- | --- |
| Exg1-for1 | ATGTTGTTCAATATCCTGATCCT |
| Exg1-rev1 | TCACCACCATATTTACCAAAGAT |
| Exg1-for2 | CTTTGGAATGGGCCAAACA |
| Exg1-rev2 | CCATCATATCTAGCCCCTGT |
| Exg1-for3 | TGAGTGGTCAGGTGCTATT |
| Exg1-rev3 | GCCTCTAAAGTAATGTTGATAATG |
| Exg2-for1 | GCCATGCTTATTTCAACTTTTATC |
| Exg2-rev1 | GTATTATCACCATTTTGGAATTCA |
| Exg2-for2 | TGATTTACATGGTGCTCCAG |
| Exg2-rev2 | GTCATTGCAGCTGACCAT |
| Exg2-for3 | CCAAACTTGGGACTATTTCAATG |
| Exg2-rev3 | GCCTTAATCACATTGACCAGG |
