# Supplementary material for: A Wickerhamomyces anomalus Killer Strain in the Malaria Vector Anopheles stephensi
Source: PLoS One. 2014 May 1;9(5):e95988. doi: 10.1371/journal.pone.0095988 (PMC4006841; doi:10.1371/journal.pone.0095988)
Supplement: Table S2 — Detection of Wa KT signal in mosquito organs by IFA with mAbKT4. Four cages containing mosquitoes fed with: (i) Sterile sugar solution, (ii) sugar solution enriched with WaATCCC 96603 (WaKT-producing strain), (iii) sugar solution enriched with WaF17.12 and (iv) sugar solution enriched with WaUM3 (WaKT non-producing strain). Organs were dissected 10, 20 and 27 days after yeast introduction and analyzed using a fluorescence microscope. (DOC) [file pone.0095988.s009.doc]

**SUPPORTING INFORMATION**

**Table S2. Detection of *Wa*KT signal in mosquito organs by IFA with mAbKT4.** Four cages containing mosquitoes fed with: (i) Sterile sugar solution, (ii) sugar solution enriched with *Wa*ATCCC 96603 (*Wa*KT-producing strain), (iii) sugar solution enriched with *Wa*F17.12 and (iv) sugar solution enriched with *Wa*UM3 (*Wa*KT non-producing strain). Organs were dissected 10, 20 and 27 days after yeast introduction and analyzed using a fluorescence microscope.
